# Water‒soil-air‒plant mutual feedback mechanism under the application of red bed composite polymers

**DOI:** 10.1371/journal.pone.0310172

**Published:** 2024-10-01

**Authors:** Tianpeng Chen, Guangjun Cui, Cuiying Zhou, Zhen Liu

**Affiliations:** 1 Guangdong Engineering Research Centre for Major Infrastructure Safety, Sun Yat-sen University, Guangzhou, China; 2 Institute of Estuarine and Coastal Research/Guangdong Provincial Engineering Research Center of Coasts, Islands and Reefs, School of Ocean Engineering and Technology, Sun Yat-sen University, Guangzhou, China; Kobe University: Kobe Daigaku, JAPAN

## Abstract

Red bed composite polymers composed of weathered red bed soil, adhesive materials, and water-retaining materials have been applied as a new type of material for environmental restoration. However, the promotion and application of this material has been limited by a lack of understanding of its action mechanism in environmental restoration. The objective of this study is to innovatively propose a water‒soil-air‒plant mutual feedback mechanism based on this material. Therefore, water‒soil-air‒plant mutual feedback tests were conducted in this study under 3 initial water contents and 10 red bed composite polymers ratios. Key parameters, namely, water content, soil conductivity, pH, temperature, O_2_ and CO_2_ contents, pigeon pea (Cajanus cajan) germination number and plant height were monitored and analyzed. As the results, a mutual feedback mechanism driving water retention, soil consolidation, air retention, and plant rooting was revealed under the application of red bed composite polymers. And, suitable environments and optimal compositions for this material are proposed. The study results provide a theoretical basis for the large-scale application of red bed composite polymers.

## 1. Introduction

Environmental restoration is the process of promoting the secondary succession of vegetation through human-initiated activities [1–5]. It is an important step in sustainable human development [6, 7]. Utilizing functional materials to enhance soil and water conservation and vegetation growth effects is an important mean of environmental restoration [8, 9]. Based on comprehensive considerations of environmental restoration effects and cost, red bed composite polymers made of weathered red bed soil, adhesive material, and water-retaining material were developed [10], and began to be applied in environmental restoration. These low cost, naturally degradable composite polymers can improve the water retention capacity of soil masses, agglomerate soil particles, improve soil structure, and enhance soil strength, thus offers good economic and environmental benefits [11, 12]. However, the action mechanism of the red bed composite polymers in environmental restoration is unclear, especially their impact on the water‒soil-air‒plant mutual feedback mechanism is not well studied, making it difficult for the polymers to be widely used in environmental restoration. Therefore, understanding the water‒soil-air‒plant feedback mechanism based on the red bed composite polymers is of great significance for their increased application in environmental restoration.

The environmental restoration is based on the interaction between water, soil, air, and plant (*eg*., [13–17]). Under the combined effects of water, soil conditions, and air content, plants can grow at their optimal state [18]. In nature, water‒soil-air‒plant mutual feedback is not always optimal. Due to the influence of external environmental factors, mutual interactions can be altered, disrupting ecosystem balance and hindering environmental restoration [19]. Water content is a key environmental factor affecting the species composition of each layer of communities in the process of environmental restoration [20]. The environmental restoration is also limited by soil conditions [21–23]: Plant reduces the incidence of net solar radiation and has a certain regulatory effect on soil temperature, resulting in daily temperature fluctuations and reduced evaporation [24, 25]; Conductivity and pH of soil play the decisive roles in ion exchange between plants and soil [26]. Changes in air content (such as O_2_ and CO_2_) affect the biochemical reaction rate during environmental restoration process [27]. Germination rate and height of plants are important indicators for regulating the evolution of ecosystems [28]. Moreover, soil microorganisms can serve as an organic medium to regulate the environmental restoration n process by reshaping bacterial communities [29, 30]. The above studies have elucidated their roles in environmental restoration from the perspectives of water, soil, air, and plant. However, further research is needed on the water‒soil-air‒plant mutual feedback mechanism based on red bed composite polymers.

Most studies have mainly focused on the effect of red bed composite polymers. For example, Zhou et al. [31] studied the quantitative relationship between soil conductivity and environmental temperature, as well as the concentration of composite polymers, and analyzed the effect of different concentrations of composite polymer addition on the growth status of small flowers and pigeon pea (cajanus cajan). Huang et al. [32] analyzed the effects of red bed composite polymers on the water retention and erosion resistance of weathered silty clay, as well as plant growth, by studying the ratio of water-retaining materials and adhesive materials. Zhou et al. [33] studied the role of water-retaining materials through shear, permeability, porosity, and plant growth experiments, indicating that they can increase the permeability, porosity, and water retention capacity of weathered red bed soil, promote plant root growth, but reduce the the shear strength of the soil. Overall, the composites enhance soil water retention and stability, form an environment conducive to vegetation survival, and help vegetation quickly recover and grow. The underlying principle in the water‒soil-air‒plant mutual feedback model of environmental restoration, is that the water-retaining material mainly drives the regulation of soil water content. When there is abundant water, the water-retaining material absorbs water and expands, while when there is insufficient water, the material releases water and contracts. Also, the adhesive materials is configured to help agglomerate soil particles, maintain soil structure, and improve soil properties, thereby helping the plants adapt to different environments [34, 35]. The above studies analyzed the role of red bed composite polymers and obtained their basic properties and environmental restoration effects. However, these studies have not clearly revealed the water-soil-air-plant feedback mechanism and the suitable application environment and optimal composition of the red bed composite polymers.

Therefore, to address the aforementioned scientific issues, we examined the effects of 10 different ratios of red bed composite polymers and 3 initial water contents on plant growth. the objectives of this study were to: (1) Determine the effects of red bed composite polymers on single factors such as water, soil, air, and plant; (2) Determine the impact of red bed composite polymers on the mutual feedback trend and mechanism of water-soil-air-plant; (3) Determine the suitable environment and optimal composition of the red bed composite polymers.

## 2. Methods

### 2.1. Experimental principles

**Fig 1:**
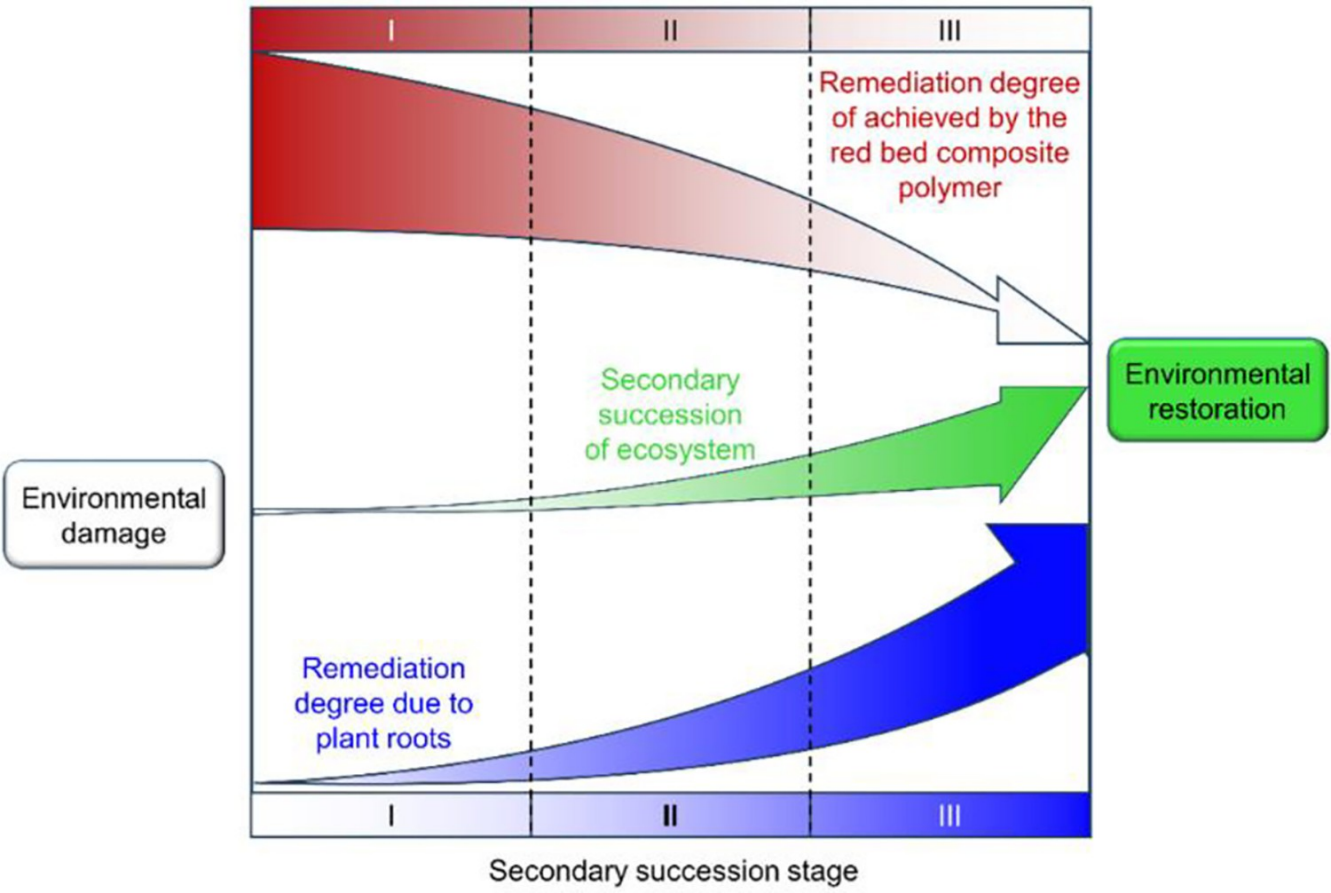
Secondary succession model of an ecosystem.

The secondary succession model for environmental restoration is shown in Fig 1 (modified from the literature [36]). According to the process of secondary succession, environmental restoration with the red bed composite polymers and plant roots can be divided into three stages [36]: (1) Stage I: Red bed composite polymers—initiated restoration stage. The plant root system has not yet developed, and restoration mainly depends on the remedial effect of composites on the soil. The adhesive material promotes soil aggregation, improves soil erosion resistance, and slows the migration of soil with water flow; the water-retaining material efficiently conserves water, enhances water-retaining capacity, slows soil erosion, enhances nutrient and air flow in soil pores, and provides environmental conditions conducive to plant growth and development [37]. (2) Stage II: Coremediation stage involving the red bed composite polymers and plant roots. Plant roots begin to grow and have a certain anchoring effect on shallow soil layers; the red bed composite polymers continue to have a significant impact on soil improvement. The growth of plant leaves increases vegetation coverage over the soil, weakens the transpiration of soil water, weakens erosion due to rainfall, and regulates the water and air content in soil pores. (3) Stage III: Plant root—driven restoration stage. The red bed composite polymers decompose until degradation is complete, and at this time, the main environmental restoration work is carried out by plant roots. Plants enter the mature stage of growth, and root systems further expand and reach deeper soil layers. Vegetation growth is abundant in various layers, and species richness increases after communities are formed. The soil fixation effect of plant roots and the hydrological effect of plant stems and leaves are enhanced [38, 39]. Ultimately, the depth and breadth of plant roots meet the requirements for environmental restoration, and plant ecosystems can effectively play a role in preventing wind and sand erosion, maintaining soil and water, conserving water sources, and promoting the favorable cycling of regional ecosystems.

In Fig 1, the degree of restoration achieved by the red bed composite polymers can be represented the aggregation and water retention induced by this material in the soil. The degree of restoration due to plant roots represents the ability of plant roots to stabilize soil during the process of root elongation and densification. Secondary succession of ecosystems indicates the carrying capacity of ecosystems in the process of environmental restoration. The red bed composite polymers are used to improve soil, in which the soil serves as a host for water‒soil-air mutual feedback, and plants, water, and air are interconnected with each other through soil as the medium. Plants grow on the soil, and plant roots absorb soil water and carry out air exchange, gradually weakening the effect of the red bed composite polymers. It can be seen that in the water‒soil-air‒plant mutual feedback model, the red bed composite polymers mainly play a role in Stages I and II of secondary succession during environmental restoration.

### 2.2. Experimental materials

**Fig 2:**
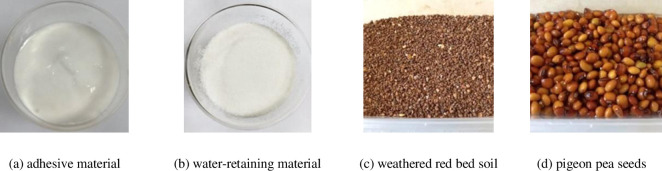
Test materials.

The materials used in this study mainly consisted of natural weathered red bed soil, a water-soluble adhesive material (gel shape), a water-retaining material (granular shape) with extremely high water absorption capacity, water, and pigeon pea seeds (Fig 2). Natural weathered red bed soil is obtained from the surface particles of weathered red bed mudstone in southeastern China, with a particle size range of 1~3 mm. Specific information on the adhesive (polyvinyl acetate, (CH_2_CHCOOCH_3_)_n_) and water-retaining (sodium polyacrylate, (C_3_H_3_NaO_2_)_n_) materials used in this study has been provided in previous studies [40]. The experimental water is tap water. Pigeon pea seeds were collected in October 2022 and are of good quality and high germination rate. The total growth time of pigeon pea is 80 days, with the first 14 days being the germination stage, the next 13 days being the seedling stage, and the last 53 days being the withering stage of pigeon pea (a decrease in watering frequency causes the natural death of pigeon pea).

### 2.3. Experimental layout

**Fig 3:**
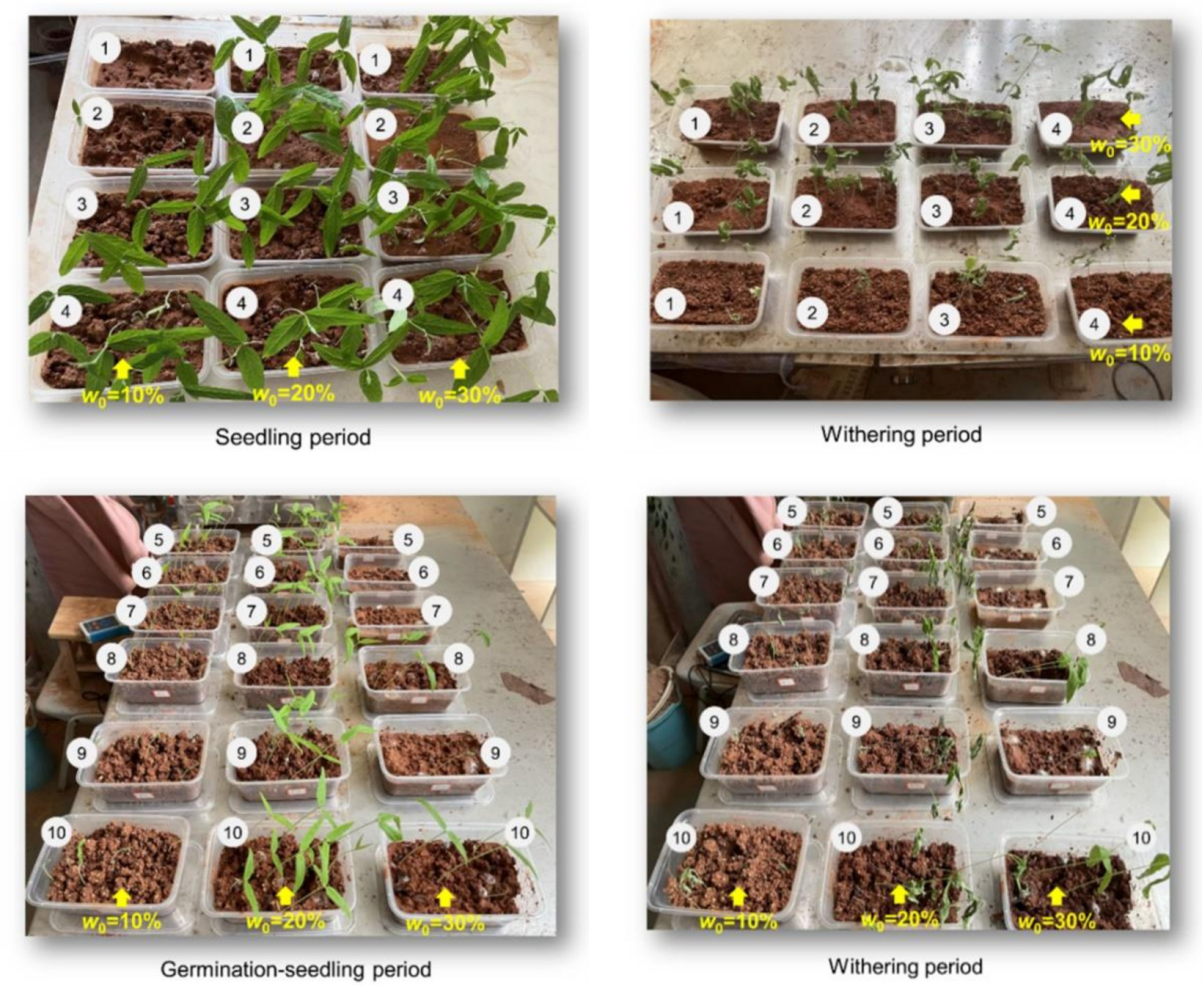
Test process (the black numbers in the figure represent the test numbers corresponding to Table 1).

Research has shown that when the adhesive material dosage is 10~15 g/m^2^ and the water-retaining material dosage is 60~70 g/m^2^, the environmental restoration effect is better [41]. Based on this, plant growth tests were conducted with different red bed composite polymer compositions (Table 1, 0.5~2.0 times the optimal content proposed by previous researchers) and initial water content conditions were designed. No. 1~4 groups are used to study the influence of different materials, while No. 4~10 groups are used to study the influence of material contents. The soil initial water contents *w*_0_ for all groups are set to 10% (in a water-deficient state), 20% (in a water-containing state), and 30% (in a water-rich state) to study the effect of water content on the water‒soil-air‒plant mutual feedback mechanism. A total of 90 plant growth tests were conducted (10 red bed composite polymer compositions × 3 initial water content × 3 replicates). The images of No. 1~10 groups during the tests at different growth stages are shown in Fig 3.

**Table 1:** Red bed composite polymer compositions.

No.	Compositions (g/m^2^)		No.	Compositions (g/m^2^)	
	Adhesive material	Water-retaining material		Adhesive material	Water-retaining material
1	0	0	6	5	60
2	10	0	7	5	30
3	0	60	8	10	120
4	10	60	9	20	60
5	10	30	10	20	120

The experimental steps include: (1) Prepare a biodegradable soil container (length of 17.5 cm, width of 12 cm, and height of 7 cm) with holes drilled at the bottom to ensure that the container is permeable and allows air entry. Sodium polyacrylate was used to prepare water-retaining material particles (the specific steps are described in the literature [40]), and the corresponding mass of this material was weighed according to Table 1. Weigh 500 g of natural weathered red bed soil, mix it evenly with the water-retaining material, and place them in a biodegradable soil container. (2) Soak seeds for 12 hours, then uniformly bury 12 seeds at a depth of 1.5 cm from the soil surface. (3) The adhesive material is prepared using polyvinyl acetate, and the specific steps are described in the literature [40]. Weigh a certain amount of this material according to Table 1, add 100 mL of water to prepare the dispersion liquid, and evenly spray it on the soil surface. Then, take 50 mL of water, spray it on the surface of the soil and leave the soil undisturbed for 2 hours. After the soil is fully saturated with water, monitoring the water content to ensure that *w*_0_ of the sample is 10%, 20%, or 30%. Finally, YT-WSYP four-in-one soil detector (see Section 2.4) was used to monitor the water content *w* in real-time throughout the experiments. By regularly adding water, ensure that *w* = *w*_0_ (with an error of less than 10%) during the first 14 days (germination stage) of the experiment. And water the samples every 15 days until *w* = *w*_0_ in seedling and withering stages. (4) According to the suitable growth environment for pigeon pea [42], place the sample in an environment with a temperature of 25°C, relative humidity of 50% RH, and light intensity of 10000 Lx.

Based on the impact of water, soil, air, and plant on environmental remediation in Introduction, the following key parameters were selected for monitoring: (1) Water characterization: water content *w*, which directly affects energy exchange between roots and soil; (2) Soil characterization: soil temperature *T*, which affects the activity of biological enzymes; ion concentration (conductivity *σ*), which affects osmotic pressure; and pH value, which affects soil fertility; (3) Air characterization: O_2_ or CO_2_ content *c*, which affect root respiration; (4) Plant characterization: germination number *n* and plant height *h*, which describe plant growth. These key parameters were recorded every two days during the germination (first 14 days) and intermittently during the seedling stages (next 13 days) and withering stage (last 53 days).

### 2.4. Experimental equipment

**Fig 4:**
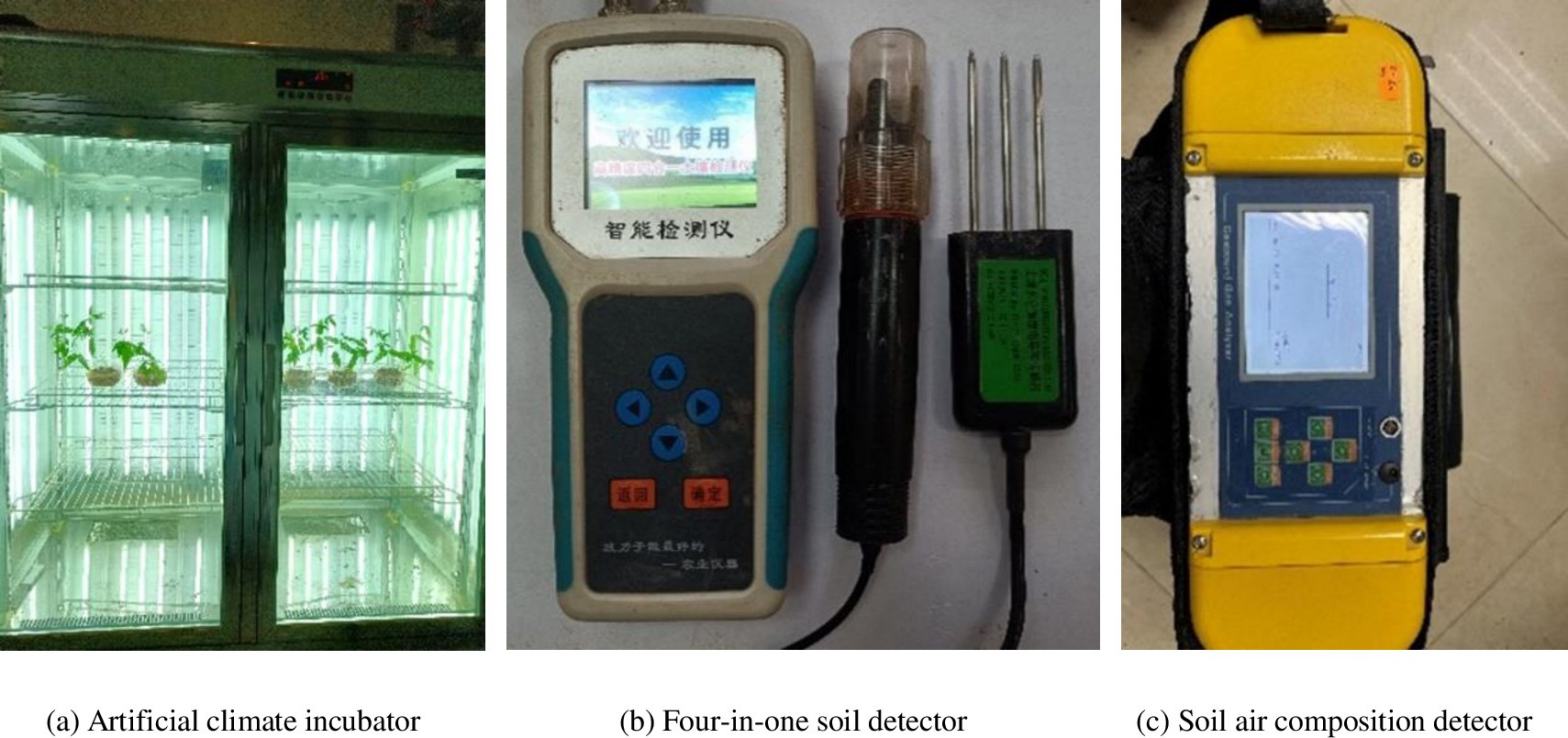
Test equipment.

The equipments used in this study (Fig 4) mainly included (1) RGX-1000 artificial climate incubator to ensure the optimal germination temperature for pigeon pea. Its temperature control range is 5 ~ 65°C, with an accuracy of 0.1°C. The humidity range is 30 ~ 95% RH, with an accuracy of 0.1% RH. The lighting control range is 0~20000 Lx, with 8-level adjustable. (2) YT-WSYP four-in-one soil detector is used to monitor the water content, soil temperature, soil electrical conductivity, and pH value. Its temperature measurement range is -40~100°C, with an accuracy of 0.1°C. The measurement range of water content is 0~100%, with an accuracy of 0.1%. The pH measurement range is 0~14, with an accuracy of 0.1. The conductivity measurement range is 0~20 μS/cm, with an accuracy of 1 μS/cm. (3) The 13.05.03PRO soil air composition detector is used to monitor the content of O_2_ and CO_2_ in soil air. The O_2_ content measurement range of soil air composition detector is 0~25%V/V, the CO_2_ content measurement range is 0~50%V/V, and the measurement accuracy is 0.01%V/V. The measurement response time is 10 s. The sampling rod has a length of 80 or 120 cm and a diameter of 1.2 cm. (4) Use a graduated scale to measure the height of plants, with a measurement range of 0~20 cm and an accuracy of 0.1 cm.

### 2.5. Statistical analyses

SPSS PRO online data analysis program was used to analyze the correlation between water, soil, air, plant and experimental time, as well as the correlation of mutual feedback characteristics between water, soil, air, and plant. The analysis method used is Pearson correlation analysis. The statistical assumption is *H*_0_: *r* = 0, which means the correlation between the two columns of data is 0, while *H*_1_: *r*≠0, which means the correlation between the two columns of data is not 0. When P<0.01, it indicates that *H*_0_ is not significantly correlated, indicating a high degree of correlation between the two columns. When 0.01<*P*<0.05, the two columns are moderately correlated.

Use Origin 9.0 software to draw the relationships between water, soil, air, plant, and experimental time, as well as the mutual feedback relationships between water, soil, air, and plant. The functions used for fitting are linear function (Eq 1), exponential function (Eq 2), and Gaussian function (Eq 3):

y=ax+b (1)

where, *a* and *b* are the slope and intercept, respectively;

$$y=A_{1}e^{-x/t_{1}}+y_{0}$$ (2)

where, *y*_0_, *A*_1_, and *t*_1_ are offset, amplitude, and decay constant, respectively;

$$y=y_{0}+Ae^{-0.5\times {\left(\frac{x-x_{c}}{w}\right)}^{2}}$$ (3)

where, *y*_0_, *x*_*c*_, *w*, and *A* are offset, center, width, and amplitude, respectively.

## 3. Results

### 3.1. Effect of red bed composite polymers on the trends of water, soil, air, and plant

**Fig 5:**
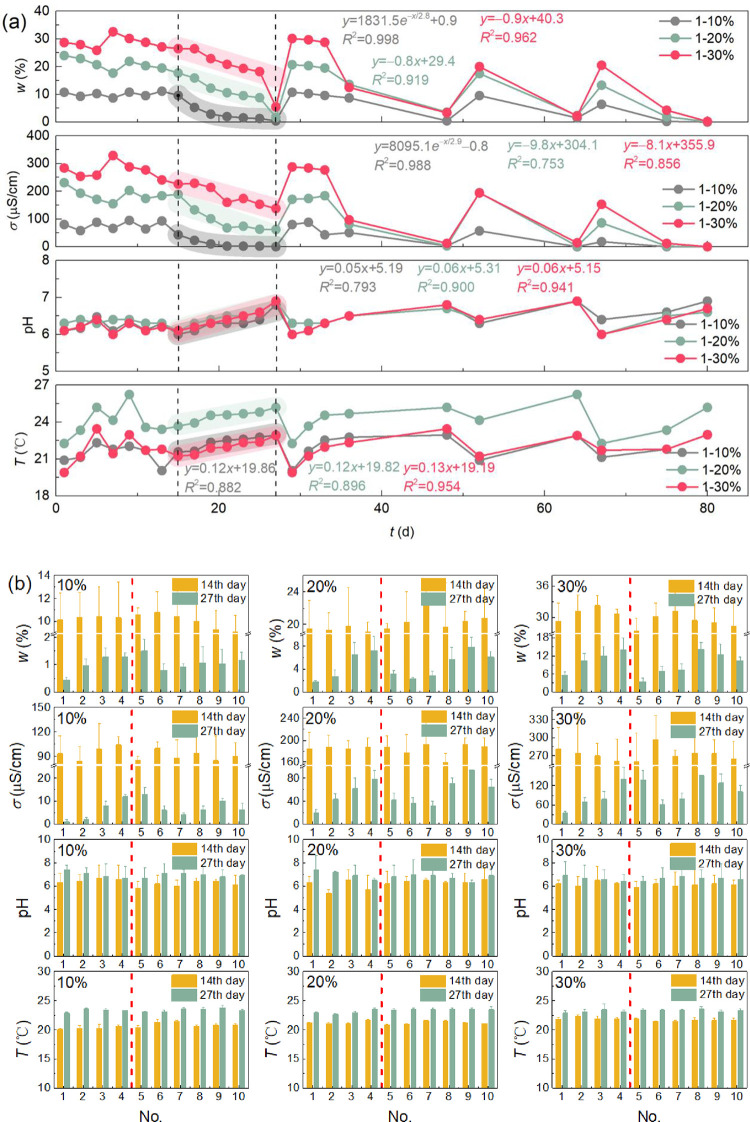
Changes in water and soil characteristics with different initial water contents *w*_0_ over experimental time *t* (a) and comparison at the beginning and ending of seedling stage (b).

The trends of water content *w*, soil conductivity *σ*, pH, and temperature *T* over *t* are shown in Fig 5A (taking No. 1 sample as an example, the trend of changes in other samples is similar to that of No. 1 sample).

Under the effect of different initial water content *w*_0_, *w* is relatively stable in the germination period, while it gradually decreases in linear functions (all are *P*<0.01) in the seedling period (*w* of the sample with *w*_0_ = 10% also decreases linearly with the experimental time from day 15 to day 20, and after day 21, *w* drops to air humidity and remains stable. Therefore, the overall trend of change shows an exponential decay law), and it fluctuates with watering time (the three peaks in the figure are the time points for adding water) in the withering period. *σ* was relatively stable in the germination period, it gradually decreased linearly ((1) *w*_0_ = 10%, *P*<0.05: from the 15th to the 20th day, the value of *σ* decreases linearly with the experimental time, and after the 21st day, it drops to air conductivity and remains stable. The overall trend of change shows an exponential decay law; (2) *w*_0_ = 20% and 30%, *P*<0.01) in the seedling period, and it fluctuated with watering time in the withering period. This trend is very close to that of *w* over *t*, indicating a correlation between *σ* and *w*. It can be seen that the trends of pH and *σ* are opposite to one another, which is consistent with the research results of Yan et al. [43] and Aini et al. [44], indicating that the nutrient content in the soil has changed, such as a decrease in cadmium content. The pH gradually fluctuates and increases over a small range over *t*. In the seedling period, pH gradually increases in a linear function (all are *P*<0.01). During the germination and seedling stages, *T* fluctuates and increases, followed stable fluctuations over a small range of *T*, and the lower *w* is, the higher the *T*. In the seedling period, *T* gradually increases in a linear function (all are *P*<0.01). There is no significant difference in the trend of changes in soil and water characteristic parameters between the beginning (14th day) and the end (27th day) of the seedling stage, as shown in Fig 5B. However, it can be seen that the No. 4 group among the No. 1~4 groups showed the best water retention, high *σ*, low pH (some other groups with pH values greater than 7 exhibit weak alkalinity, which is not conducive to the growth of pigeon pea), and a small range of *T* changes. This indicates that adding both adhesive and water-retaining materials at the same time is more conducive to water retention and beneficial for improving soil properties. Moreover, the groups that show the best water retention, high *σ*, and low pH when *w*_0_ is 10%, 20%, and 30% are No. 5, 9, and 8 in No. 5~10 groups, respectively. This means that under different initial water content conditions, different ratios of red bed composite polymers have different effects.

**Fig 6:**
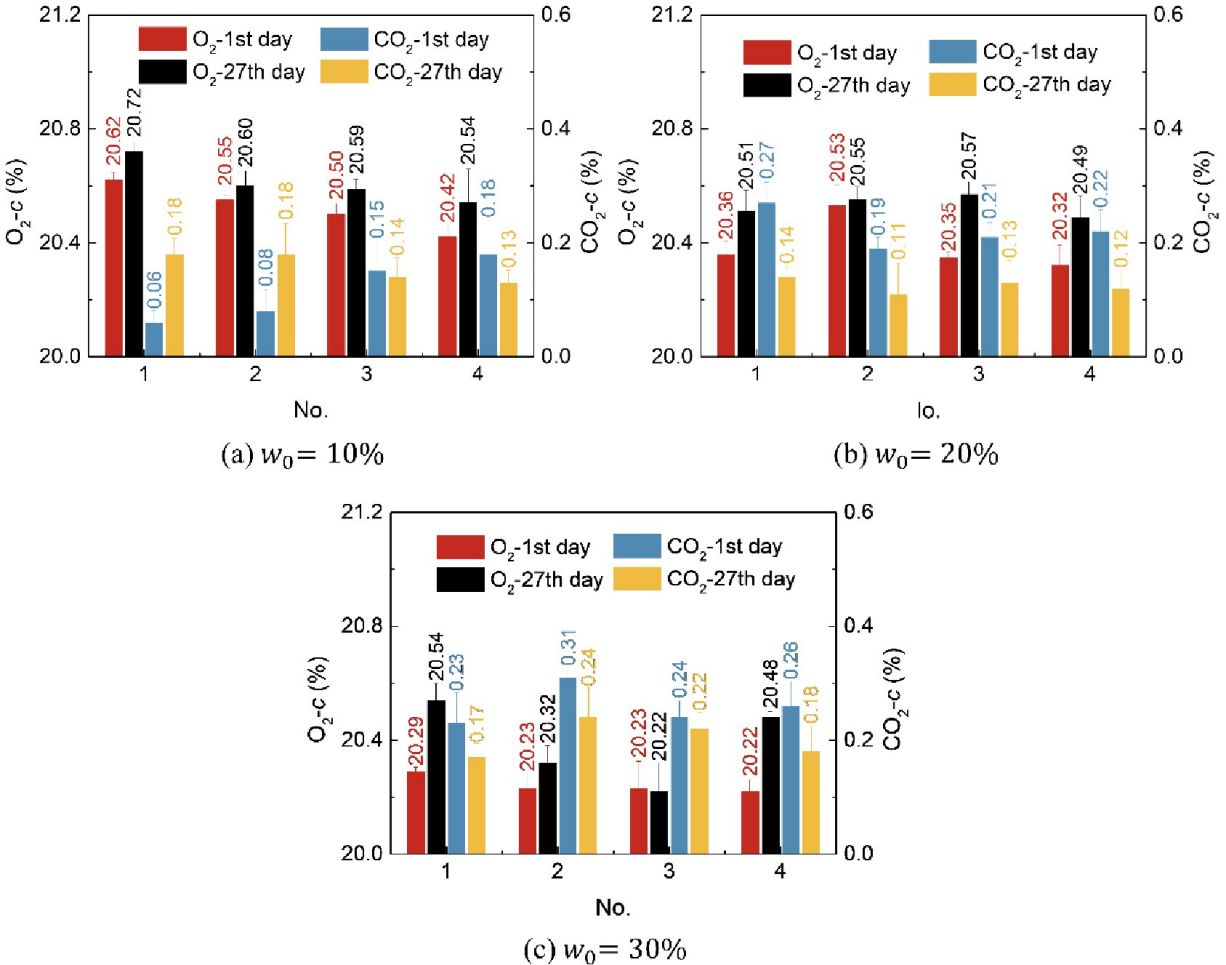
Change in O_2_ and CO_2_ content *c* over time *t* under different initial water contents *w*_0_.

In Fig 6, the O_2_ content *c* gradually increases with *t*, while the CO_2_ content *c* gradually decreases with *t*. The smaller *w*_0_ is, the higher the O_2_ content *c*; the higher *w*_0_ is, the higher the CO_2_ content *c*. In these 4 groups, the No. 4 group changed significantly in terms of O_2_ or CO_2_ content *c* (all are *P*<0.01). Previous studies have shown that the exchange of O_2_ and CO_2_ affects changes in the carbon/nitrogen ratio in organic matter reservoirs, rates of organic matter decomposition and humification, soil water and nitrogen status, soil microbial respiration, and plant rhizosphere respiration [45]. Therefore, the simultaneous addition of adhesives and water-retaining materials (No. 4 group) is beneficial for the migration of air components in the soil and for plant growth.

**Fig 7:**
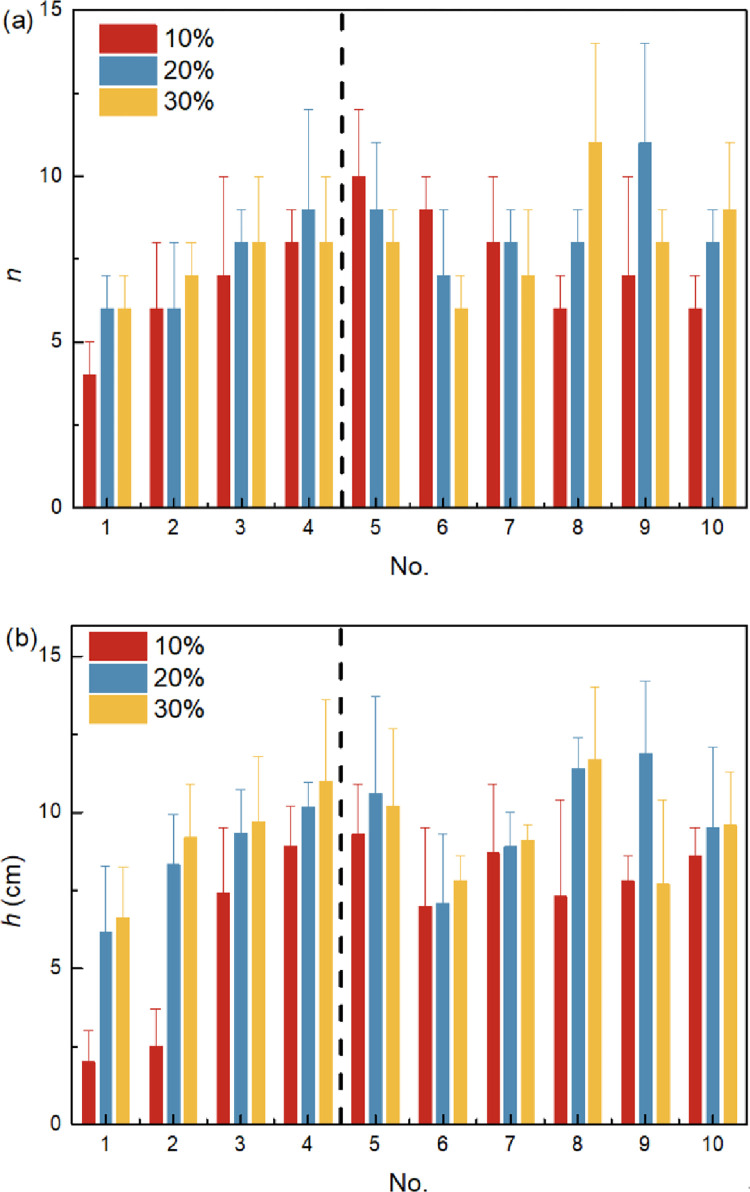
Comparisons of the germination number *n* (a) and plant height *h* (b) of pigeon pea.

Comparisons of the germination number *n* and plant height *h* of pigeon pea under the effect of different red bed composite polymer ratios and initial water contents *w*_0_ are shown in Fig 7. During the end of the germination period, the *n* of pigeon pea is the highest in No. 4 group of No. 1~4 groups, and is related to the *w*_0_ (*w*_0_ = 10%, *P*<0.05; *w*_0_ = 20% and 30%, *P*<0.01). In No. 4~10 groups, No. 5 group has the highest *n* when the initial *w*_0_ = 10% (*P*<0.05), No. 9 group has the highest *n* when *w*_0_ = 20% (no significant difference), and No. 8 group has the highest *n* when *w*_0_ = 30% (no significant difference). During the end of the seeding period, the pattern of pigeon pea plant height *h* under the effect of different red bed composite polymer ratios and initial water contents is similar to *n* in the germination period, and the higher the initial water content, the larger the plant height.

Through the above comprehensive analysis, it is indicated that the test results during the seedling stage (days 14–27) have representative significance. The summary of the test results is shown in Table 2. It was found that when *w*_0_ = 10%, the water retention effect was good, soil conductivity was high, pH changed greatly, temperature was low, O_2_ and CO_2_ content was low, plant germination rate was high, and plant height was high in the No. 4, 5, and 6 groups. When *w*_0_ = 20%, No. 4, 9, and 10 groups showed good water retention, high soil conductivity, large pH changes, high temperature, low O_2_ and CO_2_ contents, high plant germination rate, and high plant height. When *w*_0_ = 30%, No. 4, 8, and 10 group tests showed good water retention, large pH changes, high temperature, low O_2_ and CO_2_ content, high plant germination rate, and high plant height.

**Table 2:** Differences in water, soil, air, and plant of red bed composite polymers with different proportions during the seedling stage.

*w*_0_	No.	Water	Soil			Air		Plant	
		*w* (%)	*σ* (μS/cm)	pH	*T* (°C)	O_2_-*c* (%)	CO_2_-*c* (%)	*n*	*h* (cm)
10%	1	0.4	0	6.8	22.9	20.72	0.18	4	1.0
	2	1.0	0	6.7	23.6	20.60	0.18	8	2.5
	3	1.3	2	6.8	23.4	20.59	0.14	6	7.4
	4	1.3	1	6.7	23.3	20.54	0.13	9	8.9
	5	1.5	4	7.4	23.0	/	/	8	9.3
	6	0.8	6	7.0	23.1	/	/	9	9.2
	7	0.9	1	7.2	23.6	/	/	8	8.7
	8	1.1	1	7.1	23.5	/	/	6	9.3
	9	1.1	10	7.1	23.8	/	/	4	7.8
	10	1.2	1	7.1	23.3	/	/	5	8.6
20%	1	1.7	62	6.9	22.9	20.51	0.14	9	6.2
	2	2.7	78	6.5	22.7	20.55	0.11	10	8.3
	3	6.5	43	6.7	22.9	20.57	0.13	9	9.3
	4	7.2	94	6.3	23.5	20.49	0.12	9	10.2
	5	3.2	2	6.8	23.4	/	/	9	10.6
	6	2.3	6	6.9	23.5	/	/	8	11.1
	7	2.8	2	7.2	23.5	/	/	9	9.9
	8	5.6	5	7.0	23.5	/	/	6	11.4
	9	7.8	3	7.4	23.5	/	/	11	10.9
	10	6.1	5	6.9	23.5	/	/	10	9.5
30%	1	5.5	138	6.9	22.9	20.54	0.17	7	10.6
	2	10.4	128	6.1	23.1	20.32	0.31	9	9.3
	3	12.0	151	6.7	23.5	20.22	0.22	10	8.7
	4	13.9	101	6.4	23.1	20.48	0.18	9	11.1
	5	3.4	70	6.6	23.4	/	/	0	/
	6	6.9	173	6.8	23.4	/	/	0	/
	7	7.4	51	6.7	23.4	/	/	0	/
	8	14.1	48	6.6	23.6	/	/	4	8.7
	9	12.3	78	6.8	23.1	/	/	1	7.7
	10	10.4	35	6.7	23.3	/	/	4	9.6

Note: *n* and *h* are data from day 14 of the test, while others are data from day 27 of the test. “/” is data not obtained.

### 3.2. Effect of the red bed composite polymers on the mutual feedback trend of water‒soil-air‒plant

**Fig 8:**
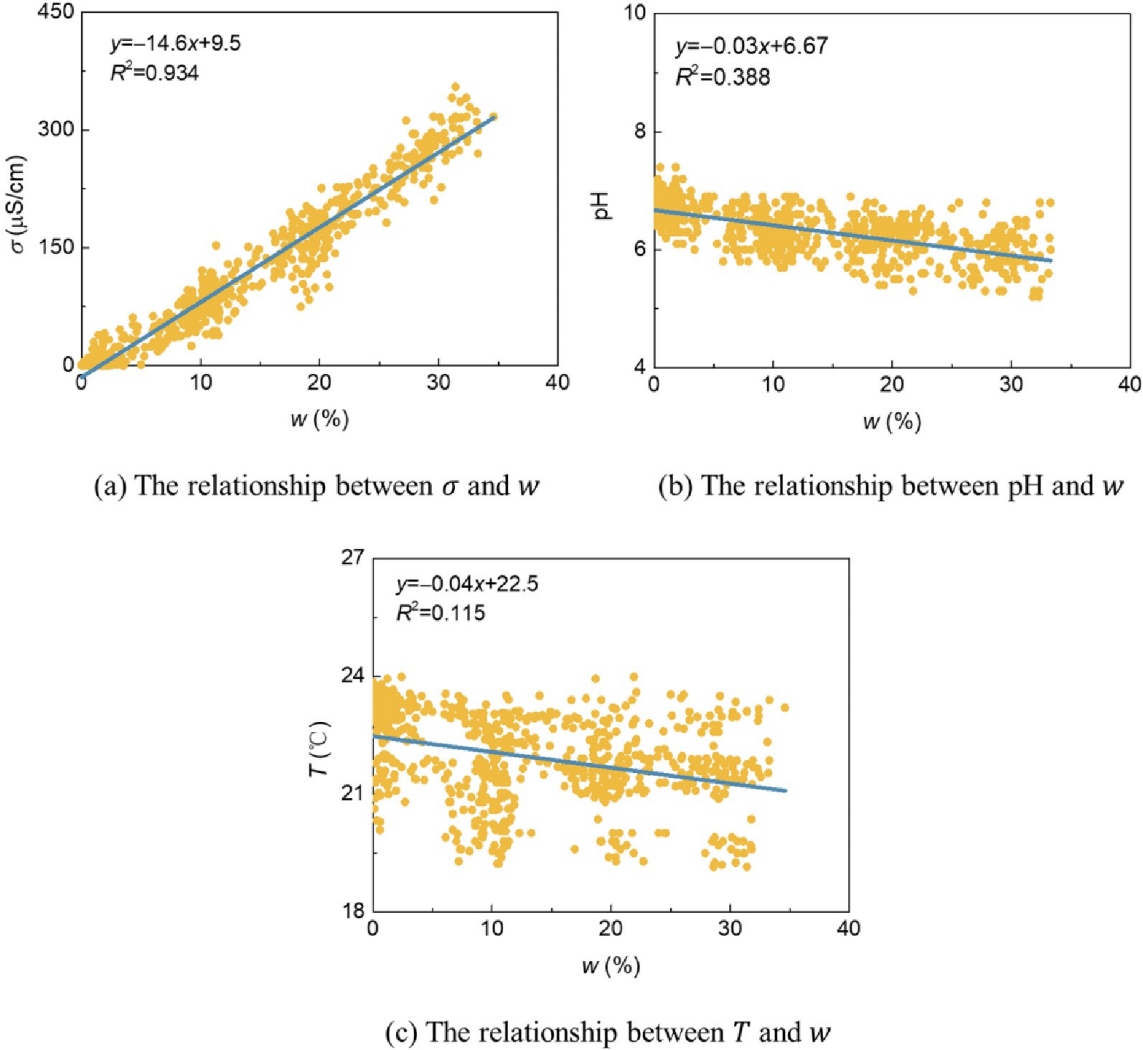
Water‒soil mutual feedback trend.

**Fig 9:**
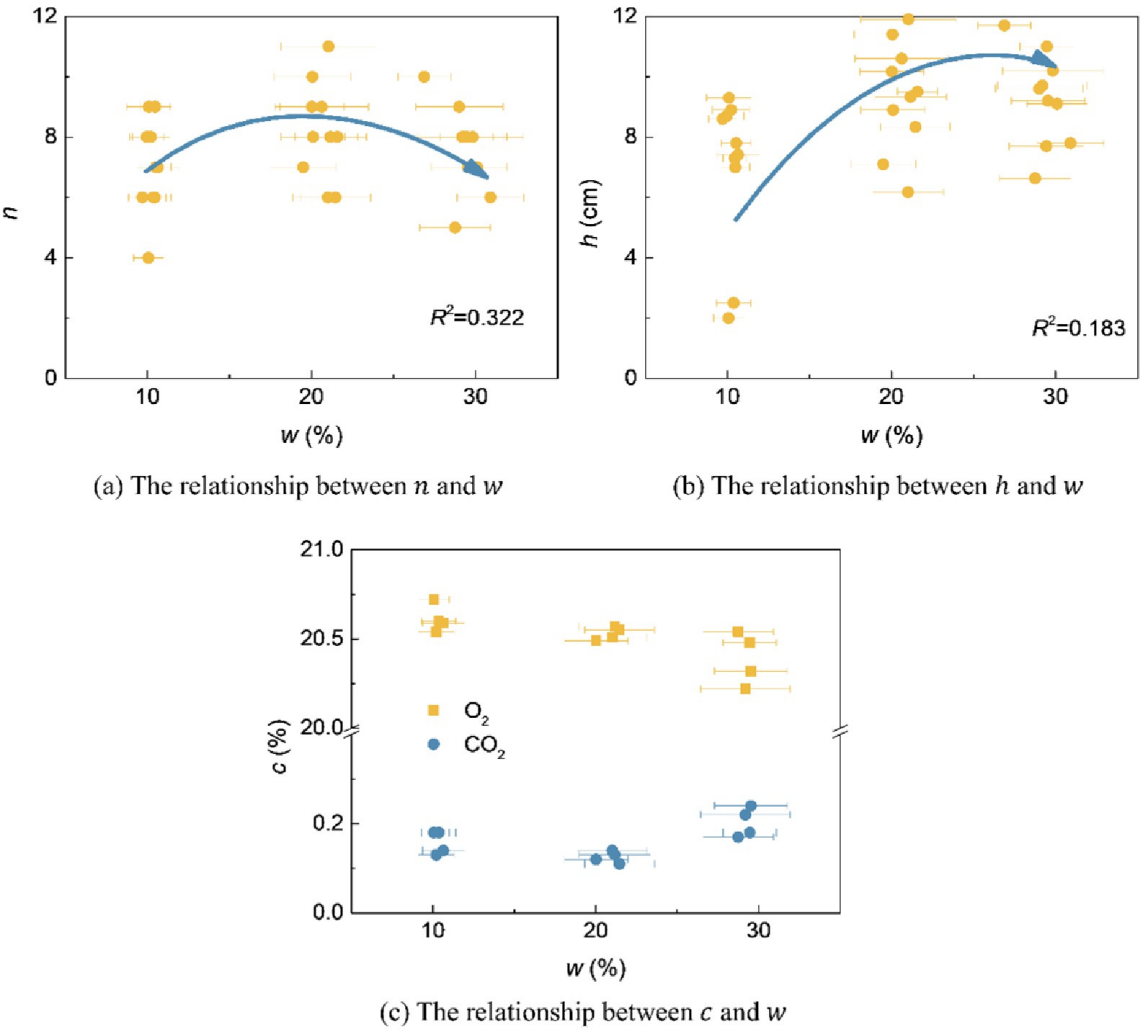
Water‒plant and water‒air mutual feedback trends.

**Fig 10:**
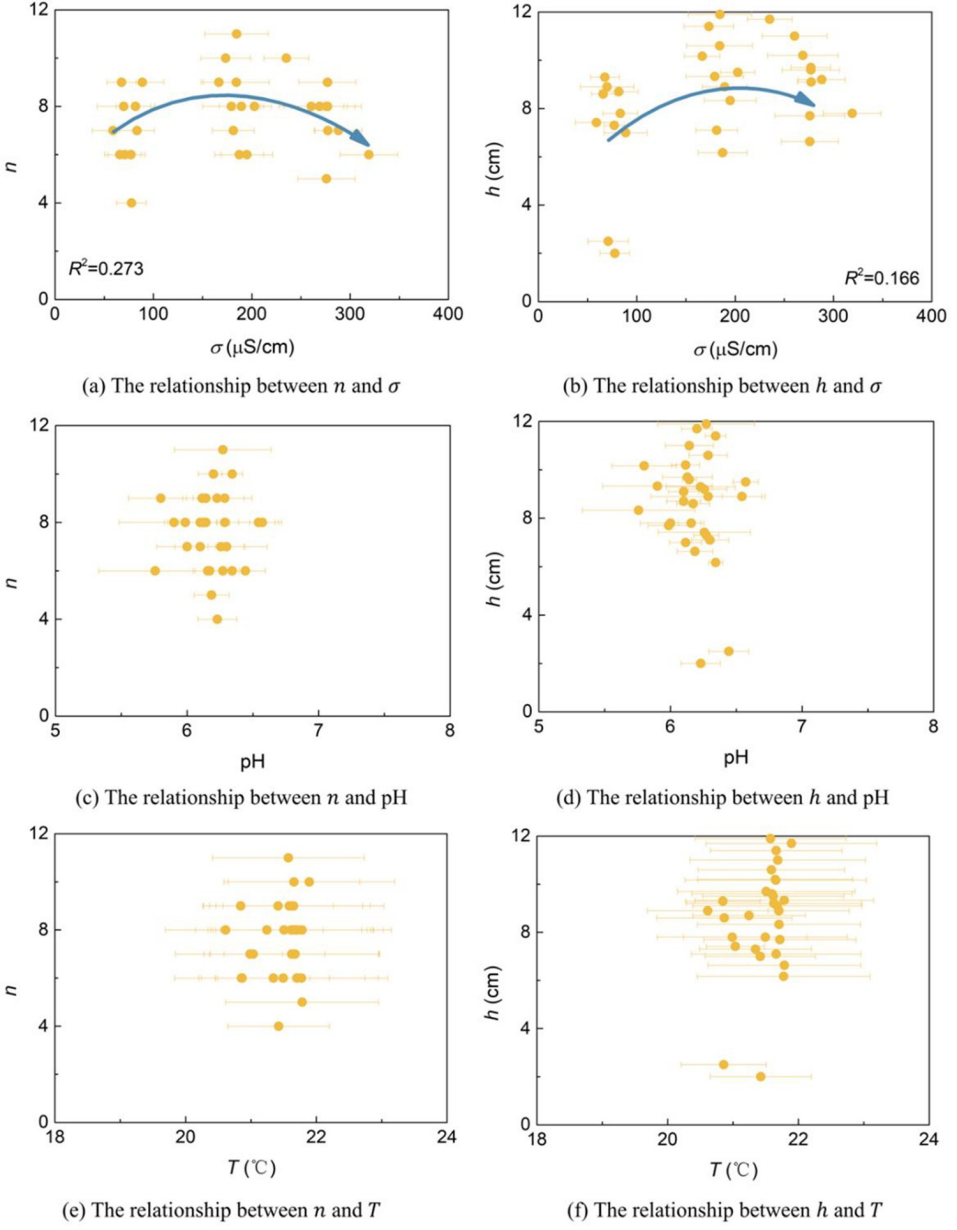
Soil‒plant mutual feedback trend.

**Fig 11:**
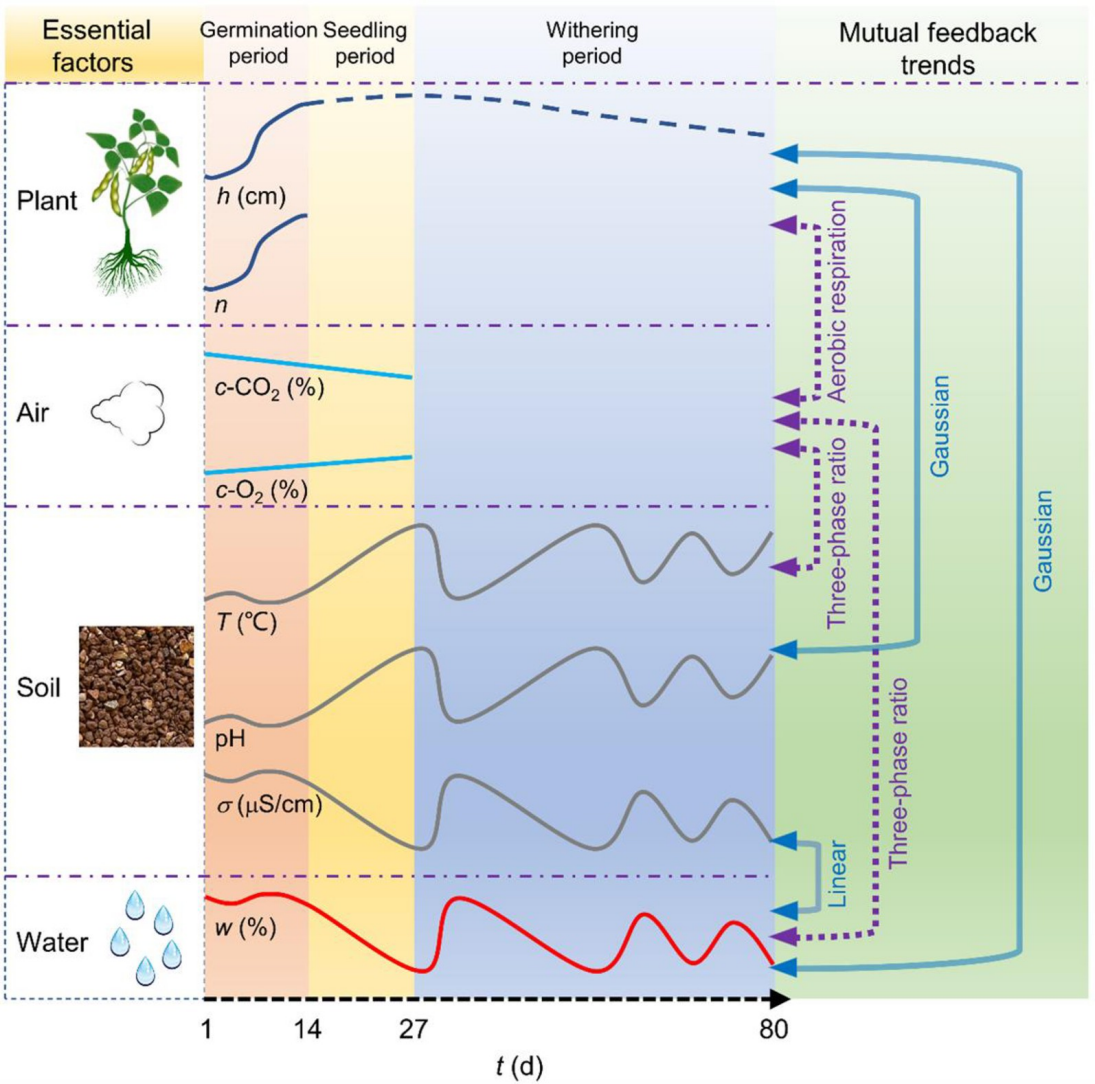
Water‒soil-air‒plant mutual feedback trends with red bed composite polymer addition.

1. Water‒soil mutual feedback trend
  The relationships between soil conductivity *σ*, pH value, temperature *T*, and water content *w* are shown in Fig 8. *σ* gradually increases with increasing *w*, and the soil pH value and *T* gradually decrease with increasing *w*. The three show a linear relationship with the change in water content (all are *P*<0.01).
2. Water‒plant mutual feedback trend
  The relationships between the *n* and *h* of pigeon pea on the 14th day and 27th day with the average water content *w* during the germination period and seeding period are analyzed, as shown in Fig 9A and 9B (all are *P*<0.01). The standard deviation of the average *w* is given. At an initial water content *w*_0_ of 20% (in a water-containing state), the plants have the highest *n* with an average of 9 (a total of 12 seeds) and the highest *h* with an average of 9.7 cm. When *w*_0_ is 10% and 30%, *n* and *h* are relatively small. The relationship between *n* and *h* with *w* was a Gaussian function (*R*^2^ are 0.322 and 0.183, respectively). Nutrient availability and microbial activity can also affect plant growth. For example, total organic carbon and total nitrogen in soil have a significant positive impact on plant growth parameters, and both total microbial biomass and major microbial groups are positively affected by total organic carbon and total nitrogen, thereby affecting plant growth [46]. According to the relationship description between the above factors, these factors do not affect the Gaussian function relationship between water content and plant growth.
3. Water‒air mutual feedback trend
  The relationships between the O_2_ and CO_2_ contents *c* of pigeon pea of No. 1~4 groups on the 27th day with the average water content *w* are analyzed, as shown in Fig 9C (all are *P*<0.01). Through analysis, it was found that as *w* increases, the O_2_ content gradually decreases, while the CO_2_ content changes less.
4. Soil‒plant mutual feedback trend
  The relationships between *n* and *h* on the 14th day and 27th day with the average soil conductivity *σ*, average pH value, and average temperature *T* during the germination period are analyzed, as shown in Fig 10 (all are *P*<0.01). The standard deviation of the average value is given. With the increase in *σ*, *n* and *h* first increase and then decrease and show a Gaussian relationship. In this study, due to the small range of pH and *T* changes, the relationships between *n* and *h* with pH and *T* could not be established. Subsequent mechanism analysis is conducted based on experience and relevant literatures.
5. Water‒soil-air‒plant mutual feedback trend
  The trends and mutual feedback relations of water‒soil-air‒plant are shown in Fig 11. Comprehensively considering the key parameters for water, soil, air, and plant during environmental restoration: In the germination period, stable soil water content *w*, soil conductivity *σ*, pH value, and temperature *T* are maintained, slight increases in the O_2_ content *c* in the soil are observed, slight decreases in the CO_2_ content *c* are seen, and the germination number *n* and plant height *h* increase. In the seedling period, the cessation of watering results in a decrease in *w*, a decrease in *σ*, an increase in pH and *T*, a slight increase in O_2_ content *c*, a slight decrease in CO_2_ content *c*, and a slow increase in *h*. In the withering period, *w* is lower than the initial water content *w*_0_ for a long time, and *σ*, pH, and *T* fluctuate greatly, resulting in a gradual decrease in *h*.

On the one hand, under the action of the red bed composite polymers, the soil can maintain an appropriate *w*. Inorganic salts dissociate into ions and dissolve in water, indicating *σ* [47]. And affect the soil pH [48], making it slightly acidic. *T* shows small fluctuations with the various physical and chemical reactions [49]. Moreover, the three-phase ratio of the soil is appropriate, and there is sufficient air (such as O_2_ and CO_2_) in the soil pores. At this point, the *w*, soil inorganic salt content, soil acidity and alkalinity conditions, and soil O_2_ conditions are all in a suitable state to support plant growth. On the other hand, plants absorb CO_2_ for photosynthesis to form organic matter and O_2_. Organic matter is transported to the plant roots within the plant body and undergoes aerobic respiration to produce CO_2_ [50]. Throughout the process, the soil O_2_ content *c* slightly increases, and the CO_2_ content *c* slightly decreases. Plant roots can fix soil and retain water and maintain soil structure without water loss, and the CO_2_ produced by the roots makes the soil slightly acidic after it dissolves in soil water [51], which together ensure the stability of *w*, soil inorganic salt content, soil acid‒base conditions, and soil O_2_ conditions. Based on this, *w* plays a dominant role in the water–soil–air mutual feedback trends.

The water‒soil-air‒plant mutual feedback trends with red bed composite polymer addition involves a process of mutual influence and dynamic stability: *w* must be dominant and sufficient, the soil must be suitable, and the air content must be sufficient to allow for plant growth. The vegetation carries out environmental restoration and undergoes secondary succession in a continuous material and energy cycle [14]. When the ratio of adhesive and water-retaining materials in the red bed composite polymers is not optimal, it leads to the formation of a poor water‒soil-air‒plant mutual feedback trend, and environmental restoration cannot be achieved. When human or natural factors cause *w* to be lower or higher than the appropriate *w* for a long time (such as during the withering period in Fig 11), the existing favorable water‒soil-air‒plant mutual feedback trend is disrupted, and the ecosystem is damaged.

### 3.3. Effects of the red bed composite polymers on the mutual feedback mechanism of water‒soil-air‒plant

**Fig 12:**
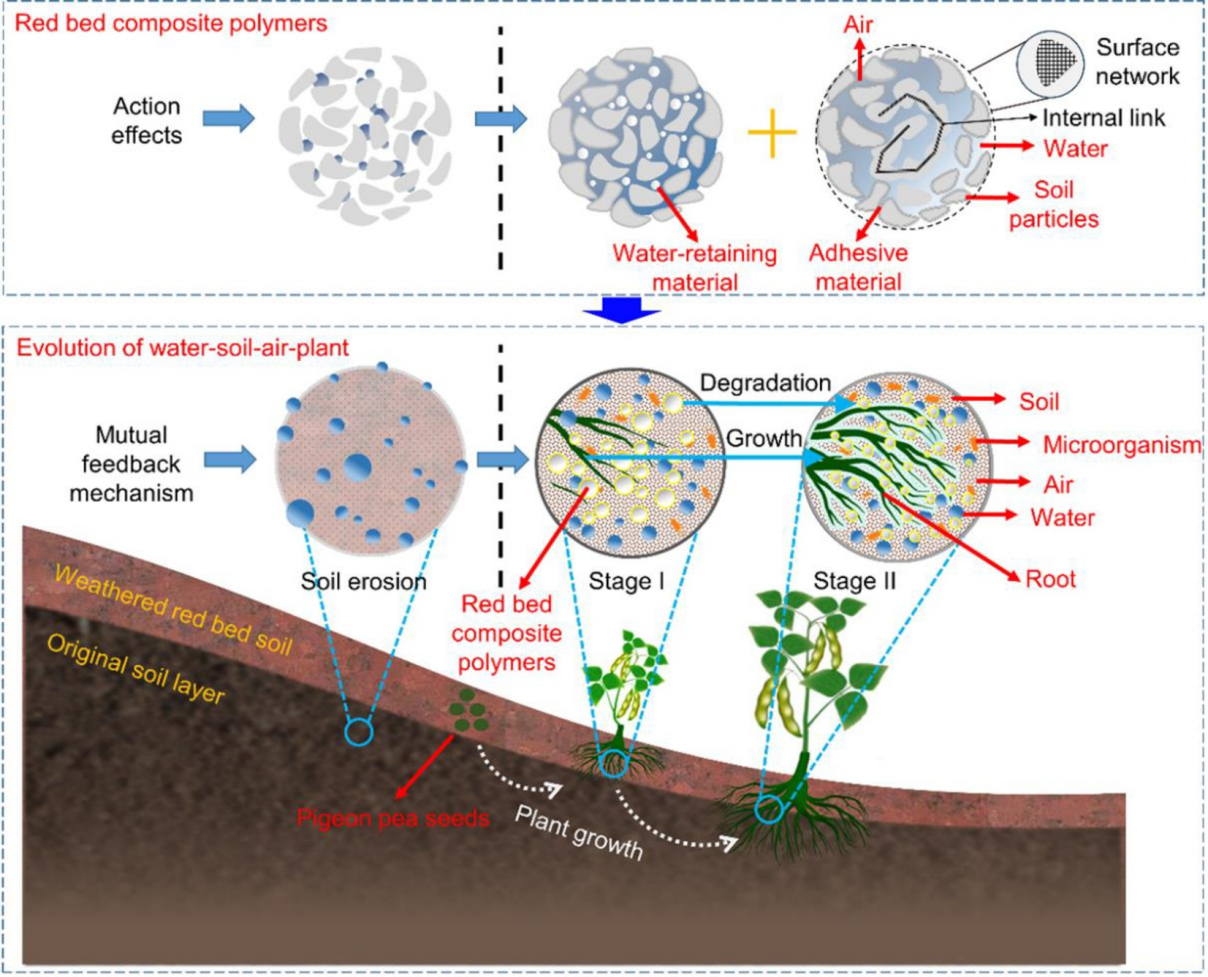
Water‒soil-air‒plant mutual feedback mechanism with red bed composite polymer addition.

The water‒soil-air‒plant mutual feedback mechanism with red bed composite polymers addition is shown in Fig 12. The mutual feedback mechanism can be explained by the direct or indirect effects of adhesive and water-retaining materials on the 4 key elements of water, soil, air, and plants. The active functional groups of adhesive materials can form hydrogen bonds with -NH_2_, -COOH, -OH, etc., in soil particles, causing soil particles to bond and form a soil aggregate structure: the surface consists of a network membrane structure, and the interior is a chain structure [52]. This structure can ensure favorable mutual feedback for water infiltration, soil stability, air diffusion, and plant respiration. This structure is elastic to a certain extent. When the water content is very low for a long time, the structure loses elasticity, and the soil solidifies. When the water content is very high for a long time, the internal chain-shaped particles absorb water and expand, producing an expansion force that exceeds the strength of the surface network membrane structure. At this point, the surface network–internal link structure based on the adhesive material is destroyed, leading to changes in water infiltration, soil structure, air content, and plant growth environment, and the favorable mutual feedback process is disrupted. The long chain polymer molecules in the water-retaining material can combine with water molecules to form a transparent hydrogel upon water absorption and release, and the material’s theoretical water absorption is about 250% [40]. This material can ensure favorable mutual feedback for water content, soil nutrients, air migration, and plant nutrient absorption. However, this ability of the transparent hydrogels is limited. When the water content is very low for a long time, the material’s water release ability reaches its limit, the soil water content decreases, and the plant receives insufficient water and nutrients. When the water content is very high for a long time, the water absorption capacity of the material reaches its limit, the soil becomes saturated, the air content in the pores decreases, and the plant receives insufficient O_2_ and CO_2_. At this point, the water absorption and release capacity of the water-retaining material is disrupted, leading to changes in soil water content, soil nutrients, air content, and the plant growth environment, and favorable mutual feedback is disrupted. And under natural conditions, adhesive and water-retaining materials degrade within 24~36 months, with the final products being CO_2_, H_2_O, and sodium salts, which are harmless to the environment [40]. This is different from the ecological risks caused by materials such as polyethylene microplastics and film residues [53, 54].

Based on the specific interactions between the red bed composite polymers and soil with different initial water contents mentioned above, the soil water retention is enhanced during the ecological restoration process, resulting in high water content and soil conductivity monitored in this study. The enhanced cohesion of soil particles and stable soil porosity during the ecological restoration process resulted in high O_2_ and CO_2_ contents in this study. The plant roots fully absorbed soil nutrients under the action of microorganisms, causing the soil pH and temperature to gradually increase, resulting in more plant germination number and higher plant height. Accordingly, in the original soil layer where soil erosion occurs, the red bed composite polymers is applied, and then plants are planted. In stage I of the secondary succession process, the red bed composite polymers improve the water, soil, and air environment, enabling pigeon pea to germinate and grow. In stage II of the secondary succession process, pigeon pea and corresponding microorganisms gradually replace the role of the red bed composite polymers in restoration, and plant roots infiltrate into the original soil layer and optimize its water, soil, air, and biological environment, achieving mutual feedback for water conservation, soil fixation, air holding capacity and plant rooting. The above-mentioned ecological restoration mechanism involves the water‒soil-air‒plant mutual feedback mechanism of plant-physical-chemical restoration, which is the main ecological restoration method in the process of land degradation and soil degradation restoration in China [55]. Moreover, the process of this ecological restoration mechanism can be shortened from 22 years [56] to six months to two years after the addition of red bed composite polymers.

## 4. Discussion

Based on the basic trends in water, soil, air, and plant, as well as mutual feedback among these 4 key elements, the applicability of the red bed composite polymers is summarized, and the optimal composition of these polymers is proposed.

### 4.1. Applicable environment of red bed composite polymer

The red bed composite polymers are applicable under 3 initial water content conditions. Under a water-deficient state (initial water content *w*_0_ = 10%), the water-retaining material has stronger water absorption than soil particles and has a certain water locking effect, hindering water absorption by plant roots and causing slow plant growth, but the water-retaining material can retain soil water for a long time [33]. After the adhesive material is sprayed, a dense polymer layer easily forms on the soil surface, making the soil firm and not conducive to soil breakage during the plant germination period. However, the simultaneous application of water-retaining and adhesive materials can compensate for the shortcomings of the individual materials. Compared to undisturbed red bed soil without the addition of polymers, the growth parameters of the plants are significantly improved. Under a water-containing state (*w*_0_ = 20%), after water absorption, the water-retaining material expands, expands the soil pores, increases the specific surface area of plant roots and air, and plant can better perform cellular respiration, which promotes plant growth. After the adhesive material is sprayed, it tightly connects with soil particles, forming a dense polymer film on the surface of the soil, which can effectively preserve soil water, ensure a stable soil environment for plant growth, and thereby improve the survival rate of the plants. Under a water-rich state (*w*_0_ = 30%), the water-retaining material absorbs excessive amounts of water and has a significant expansion effect, blocking soil pores and increasing the specific surface area of the air in contact with plant roots. When it absorbs excess water, plant roots do not grow Rhizobiaceae because they are in anoxic soil conditions, which inhibits nitrogen cycling and plant growth [13]. The molecular membrane of the adhesive material is prone to damage, resulting in an insignificant water-retaining effect and a rapid decrease in soil water. Therefore, it is necessary to replenish water in a timely manner.

Due to the limited length of the article, this study only includes the effects of different initial water contents and single climatic conditions on the applicable environment of red bed composite polymers. The on-site application of red bed composite polymers under special climatic conditions such as extreme cold, extreme heat, extreme drought, and extreme humidity, as well as the water-soil-air-plant mutual feedback mechanism under their influence, will continue to be studied as a future research direction. Special soil types on site, such as frozen soil, saline soil, and collapsible loess, can also have an impact on the on-site application of the water-soil-air-plant mutual feedback mechanism in the application of red bed composite polymers, which is one of the future research directions. Different ecological interactions or existing ecological degradation conditions are also one of the factors that affect the on-site application of this research result, and will also be one of the future research directions.

### 4.2. Optimal composition of red bed composite polymer

According to the test results (Table 2), under a water-deficient state, the No. 5 group in Table 1 (adhesive material: 10 g/m^2^, water retaining material: 30 g/m^2^) represents the best composition. Soil with a low water content requires less of the red bed composite polymers to achieve soil fixation and water retention. Under a water-containing state, the composition of the No. 9 group (adhesive material: 20 g/m^2^, water retaining material: 60 g/m^2^) is most suitable, and the content of the red bed composite polymers doubles, reaching deeper soil layers for soil particles aggregation and slowing soil water evaporation during the stage at which water supply was decreased. Under a water-rich state, the No. 8 group (adhesive material: 10 g/m^2^, water retaining material: 120 g/m^2^) represents the best composition. A soil with a high water content requires more water-retaining material. The internal particles are prone to water absorption and expansion, and a low content of adhesive material is more conducive to a large deformation in the molecular membrane and the fixation of soil particles.

## 5. Conclusions

1. The water‒soil-air‒plant mutual feedback mechanism under the application of red bed composite polymers for environmental restoration is unclear. Therefore, mutual feedback tests based on these polymers were carried out, and dynamic and stable mutual feedback trends corresponding to various water, soil, air, and plant parameters were observed. Mutual feedback mechanism involving water retention, soil fixation, air retention, and plant rooting is revealed under the joint action of the surface network–internal link structure based on the adhesive material and the hydrogel formed with water absorption-water release of the water-retaining material.
2. The study results indicate that key parameters such as water content, soil conductivity, pH value, temperature, O_2_ and CO_2_ contents, germination number and plant height of pigeon pea exhibit regular changes with time during the pigeon pea germination, seedling, and withering periods. The water‒soil mutual feedback presents a linear relationship, and the water‒plant and soil-plant mutual feedback present Gaussian relationships. In the red bed composite polymers, the suitable ratio of adhesive to water-retaining materials for the water-deficient state is 10 g/m^2^ and 30 g/m^2^; the suitable ratio for the water-containing state is 20 g/m^2^ and 60 g/m^2^; and the suitable ratio for the water-rich state is 10 g/m^2^ and 120 g/m^2^, respectively.
3. A suitable environment and optimal composition of the red bed composite polymer is proposed in the study, providing a theoretical basis for the large-scale application of these polymers, which can be promoted and applied to fields for agricultural soil improvement, desertification control, and landscape restructuring.
4. This study is the result of laboratory tests conducted under different initial water contents and single climatic conditions. The on-site application (scale transformation) of these achievements into environmental restoration such as land degradation and soil degradation still requires large-scale field tests for verification, which is one of our future research contents. In addition, the limitation of this study is mainly that it did not consider how different climate conditions, soil types, and ecological interactions affect the effectiveness of red bed composite polymers in real-world environmental remediation projects. These research directions need to be further expanded.

## Supporting information

S1 File (XLSX)
